# Factors impacting human-perceived visual quality on television displays

**DOI:** 10.3389/fnins.2024.1426195

**Published:** 2024-12-19

**Authors:** Eddie Pei, Hosub Lee, Elena Fedorovskaya, Susan Farnand

**Affiliations:** ^1^Munsell Color Science Laboratory, Rochester Institute of Technology, Rochester, NY, United States; ^2^Visual Solution Lab, DMS, Samsung Research America, Irvine, CA, United States

**Keywords:** video quality, ambient environment, video content, display setting, viewer characteristics, image appearance model

## Abstract

There is a growing interest in understanding the factors that influence a user's perception and preferences for video quality. This study specifically focuses on how various factors, including video content, display settings, viewer characteristics, and the ambient environment, affect the subjective video quality assessment (VQA) of TV displays. To investigate these factors, two psychophysical experiments were conducted, and the results indicate that all four factors have a significant impact on video quality perception in different ways. This study is beneficial for researchers and developers who aim to improve display and environmental settings to provide viewers with the best possible viewing experience.

## 1 Introduction

Researchers have been exploring ways to enhance video and image quality since the television became a staple in everyday life. In the early 1980s, the work by Schreiber (1984) delved into enhancing video quality by leveraging insights from the human vision system (HVS), examining how its characteristics, such as spatial and temporal vision and adaptation, influence perceived video quality. As high-definition television (HDTV) gained popularity in the early 2000s, researchers explored the interplay between viewer characteristics (e.g., gender) and image quality, concluding that the sense of presence is significantly affected by image quality (Bracken, 2005).

With the advancement in video and display technologies, the demand for high-quality video has increased markedly over recent years. In this context, assessing the video quality to ensure the optimal user viewing experience has become crucial. VQA involves analyzing various factors that affect the overall human-perceived visual quality of video content, such as image resolution, content genre, viewer characteristics, and many others. Overall, these factors are categorized into four main domains: video content, display, viewer characteristics, and ambient environment. Below we summarize representative studies in each domain.

### 1.1 Video content

Research has shown that viewer perception of image quality varies with the content. Park et al. studied how people perceive image quality differently depending on the type of content. Through the experiments, they concluded that content genre-based image quality adjustment, such as enhanced saturation for sports scenes, is necessary (Park, 2021). Some studies have also investigated the impact of the type of content on viewer preferences for video enhancement algorithms and have introduced datasets like the “LIVE” for evaluating video distortion through both subjective and objective methods (Seshadrinathan et al., 2010; Moorthy et al., 2012; Satgunam et al., 2013). Moreover, García Castiella (2020) explored the effects of the dynamic range in the video content.

### 1.2 Viewer characteristics

Studies focusing on viewer demographics have revealed insights into how such characteristics as gender and age may influence quality perception. Sinno and Bovik (2018) did an online experiment and found only minor differences in quality ratings between male and female viewers, however age did have an impact on ratings. Human vision changes with age, resulting in differences in video quality perception. In this regard, Park and Park (2018) divide the viewers into the adult group and elderly group and examine the image quality preference of the elderly group. This study confirms that it is necessary to increase the brightness of the image in order for the elderly group's image quality preference to be at an equivalent level to that of the younger adult group. Nam et al. (2005) also provide methods for adapting visual content to accommodate color vision deficiency and low-vision capabilities. These studies underscore the necessity of considering a wide range of viewer characteristics, including age and visual ability, in the optimization of image quality.

### 1.3 Ambient environment

The influence of the ambient environment on image and video quality has been a significant focus of the research area. Liu et al. (2014) discussed the effect of ambient light intensity on hand-held displays. The quantitative analysis suggests that differences in display reflection coefficients do not affect the low illumination performance of the device but rather the performance at higher levels of illumination. Wetzel and Hernandez (2010) conducted an experiment evaluating six factors related to ambient light and concluded that the intensity of ambient light significantly affects the accuracy of determining the stimuli but not the response time. Chubarau et al. (2020) delved into how ambient illumination levels and display luminance affect image quality. They found that humans preferred the illumination range near the “ideal” conditions, around 200–600 lux. They also came up with a model. These studies highlight the critical role of ambient environmental conditions, especially lighting, in viewer experience.

### 1.4 Display

The Display domain includes display settings and display technologies. Kufa et al. investigated the perceptual quality of video content presented in Full HD and Ultra HD resolutions at different viewing distances. This research highlighted VQA is directly correlated with resolution and bitrate (Kufa and Kratochvil, 2019). Baek et al. (2021) investigated how the correlated color temperature (CCT) of the TV and ambient light influences perceived image quality. Their study suggests a preference for adjusting the CCT of television displays to resemble the ambient light's CCT, noting that the optimal display intensity is typically lower than the surrounding light.

VQA also involves the use of subjective and objective methods. Objective methods use mathematical or statistical tools to analyze the technical aspects of the image or video content itself, and they are highly efficient and easy to deploy. Over the years, more advanced objective methods have been developed, including spatial domain methods such as deep similarity index (DeepSim), based on a deep neural network (Gao et al., 2017). Chikkerur et al. (2011) and Zhai and Min (2020) provided a thorough review of the objective VQA methods. The human vision system is significantly related to the perceived image and video qualities. Some researchers have connected objective methods with HVS. Varga introduced an innovative quality-aware feature extraction method for no-reference image quality assessment by applying various sequences of HVS-inspired filters (Varga, 2022). Gao et al. (2010) summarized the state-of-the-art of HVS-inspired IQA methods; they introduce the basic theories and development histories of HVS-based IQA methods in their work. Panetta et al. (2015) proposed HVS-inspired enhancement of the underwater images. However, some of these methods are generally pixel-by-pixel based and do not consider the actual user-perceived visual quality that other factors, such as ambient environment, can influence. Conversely, subjective methods rely on human observers to perceive and evaluate the quality of video content based on their personal preferences and opinions. Series (2012) is a standard that introduces subjective evaluation methods. Pinson and Wolf summarized different subjective evaluation methods while providing valuable insights into the strengths and limitations of different methodologies (Pinson and Wolf, 2003). Both objective and subjective methods can help evaluate video quality. Although it is more time-consuming than the objective method, the subjective VQA method is more suitable for measuring observers' image quality preferences while considering user-specific and situational factors.

This study extends earlier work (Pei et al., 2024). The aim is to empirically uncover and examine TV users' video quality preferences and impacting factors. Factors from four categories were carefully chosen, namely, observer characteristics, display, ambient environment, and viewing content. The analysis delves deeper into these factors, particularly focusing on observer characteristics. We investigated demographic factors, which were not previously discussed. The subjective method was adopted as the initial investigation, and the objective method was implemented to gain more insight. Additionally, a comparison of color spaces is included to enhance understanding and predictive modeling. Two psychophysical experiments were designed with different considerations of selected factors.

## 2 Experimental methodology

The goal of the experiments is to determine how users perceive picture quality on TV displays and what latent factors affect human visual perception. Toward this end, two experiments were designed to evaluate the impact of varied combinations of select factors on specific video quality, implemented by a TV display's picture setting configurations and an image processing algorithm. Both of these experiments involved the same participants, TV displays, lighting environment, and similar contents.

### 2.1 The participants

**Figure 1:**
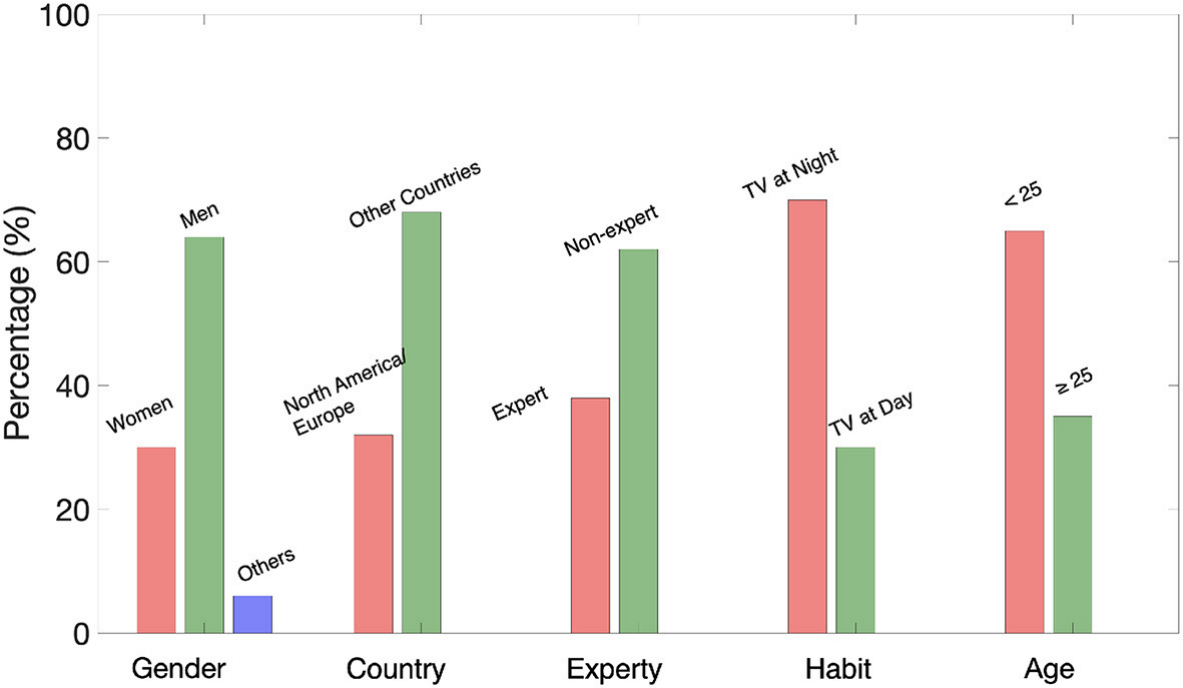
Demographic factors.

Individuals with normal color vision were recruited as participants in the experiments. Each participant provided informed consent after being briefed on the study procedures. RIT's Human Subjects Research Office has approved this experiment (approval FWA #00000731). 37 observers participated in both of the experiments. All of the participants were paid volunteers. Among all the observers, there were 30% women, 64% men, and 6% others. 32% of them were from North America/ Europe, and 68% were from other countries. 38% of the participants had domain knowledge of color or image science. 70% of them preferred watching TV during the night time. And 65% were younger than 25 years old. The demographic factors are depicted in Figure 1.

### 2.2 Experiment factors

**Figure 2:**
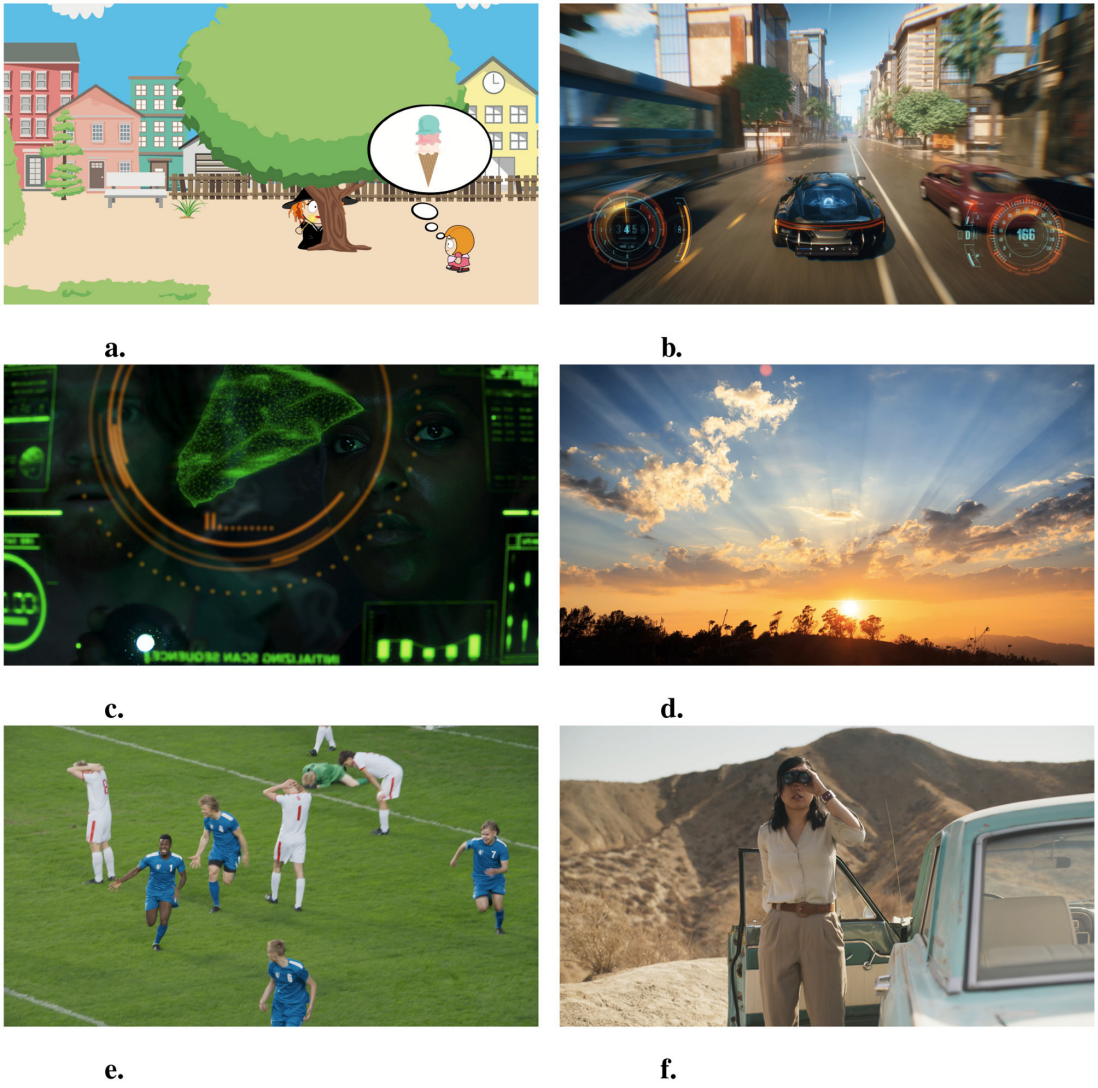
Video stimuli (content). **(A)** Animation. **(B)** Game video. **(C)** Dark movie scene. **(D)** Nature scene. **(E)** Soccer scene. **(F)** Bright movie scene. Adapted with permission from Pixabay **(A)**, American Society of Cinematographers **(C, F)**, and Adobe Stock **(B, D, E)**.

The experiments comprised evaluation of six distinct visual stimuli, as depicted in Figure 2. The contents represented various video categories, including bright and dark scenes, diverse skin tones, and animated and real-life footage. Each stimulus was prepared as an 8-second-long 4K SDR video clip per selected content. The original video format was Apple's QuickTime (MOV), and HEVC was used as an encoder for easy content playback on TVs.

The experiments were conducted in the Munsell Color Science Laboratory Dynamic Visual Adaptation Lab, which features a ceiling-mounted, five-channel tunable LED system. Various desired lighting conditions can be achieved by adjusting the weights for these channels. Under the guidance of the report from the U.S. Department of Energy (Secretary, 2012), this study employed four distinct lighting conditions: (1) Dark Warm, (2) Bright Warm, (3) Dark Cool, and (4) Bright Cool (see Table 1). The ambient lighting characteristics were measured using an MK350N spectroradiometer.

**Table 1:** Select factors.

**CCT (2)**	**Illuminance (2)**	**Stimuli (6)**	**Demographic factors (6)**	**Picture settings (5)**
2700K (warm)	15lux (dark)	Animation	Age	Dark standard mode^*^
5500K (cool)	350lux (bright)	Game	Gender	Bright movie mode^*^
		Movie (dark)	Expertise	Low colorfulness^**^
		Nature	Habit	Medium colorfulness^**^
		Sports	Country	High colorfulness^**^
		Movie (bright)		

^*^ and ^**^ were used for Experiment I and II, respectively.

The display setting was chosen as one of the factors. It is also known as Picture Mode on TV displays. In the first experiment (Experiment I), the observers were asked about their preferences regarding specific Picture Modes, which were developed based on TV usage log data (Lee and Park, 2021; Lee, 2023). These settings differed from the factory default, mainly on brightness, contrast, and sharpness levels that were actually preferred by the users. To be more specific, our log data analysis indicated that some users prefer to customize the default Standard and Movie Picture Mode. For Standard Mode, some users tend to lower the TV display's brightness level by 22%, compared to the default (namely, Dark Standard Mode). Different preferences exist for Movie Mode, like increasing the brightness setting by 37% more than the default (namely, Bright Movie Mode). We configured the default and newly identified Picture Modes on the reference and control TVs and asked subjects to mark their preferences for new Picture Modes. The second experiment (Experiment II) focused on the video's colorfulness. Here, we defined three different levels of colorfulness based on a proprietary perceptual color enhancement algorithm (Su et al., 2023) and collected subjects' preferred colorfulness levels accordingly. The display was calibrated for each mode under a dark condition to ensure accurate color rendering on the test TV.

In the experiment, the test TV implemented a different color enhancement algorithm than the reference TV, which impacted its apparent colorfulness. It was configured to show (1) low, (2) medium, and (3) high levels of colorfulness for separate trials, while the baseline colorfulness of the reference TV was held constant. The test TV's lowest colorfulness level was roughly equivalent to the colorfulness of the reference TV. Through the experiment, we aimed to determine how people react to different colorfulness levels depending on select factors.

As discussed in the Introduction, observer characteristics significantly affect the VQA and could offer insights into the individual differences in the video/image preference. Thereby, we considered five factors under the observer characteristics category for the current study. This includes (1) age, (2) gender, (3) expertise in color or image science, (4) habituation of watching TV, and (5) country of origin. The details are listed in Table 2.

**Table 2:** Demographic factors.

**Habit (2)**	**Expertise (2)**	**Country (2)**	**Gender (3)**	**Age (2)**
Night time	Color expert	North America/Europe	Men	< 25
Other time	Non-color expert	Others	Women	≥25
			Other	

### 2.3 Experiment I: procedure

Two identical 65-inch Samsung 4K QLED TVs (Model: QN85C) were installed side-by-side and used as the displays for Experiment I. The double stimulus simultaneous quality scale (DSSQS) method was adopted. The reference stimulus with default Picture Mode on the left TV was assigned a preference score of 50, and observers rated the video quality of the control stimulus with newly identified Picture Mode (e.g., Bright Movie Mode) on the right TV on a scale as low as 0 but without upper limits compared to the reference. This experiment was divided into four blocks, each representing one of the specific lighting conditions explained above. Within each block, the stimuli were presented in a random order to prevent memory effects. A MATLAB program was used to control the experimental procedures, and a Bluetooth keypad was used as the input device for observers to submit their responses.

**Figure 3:**
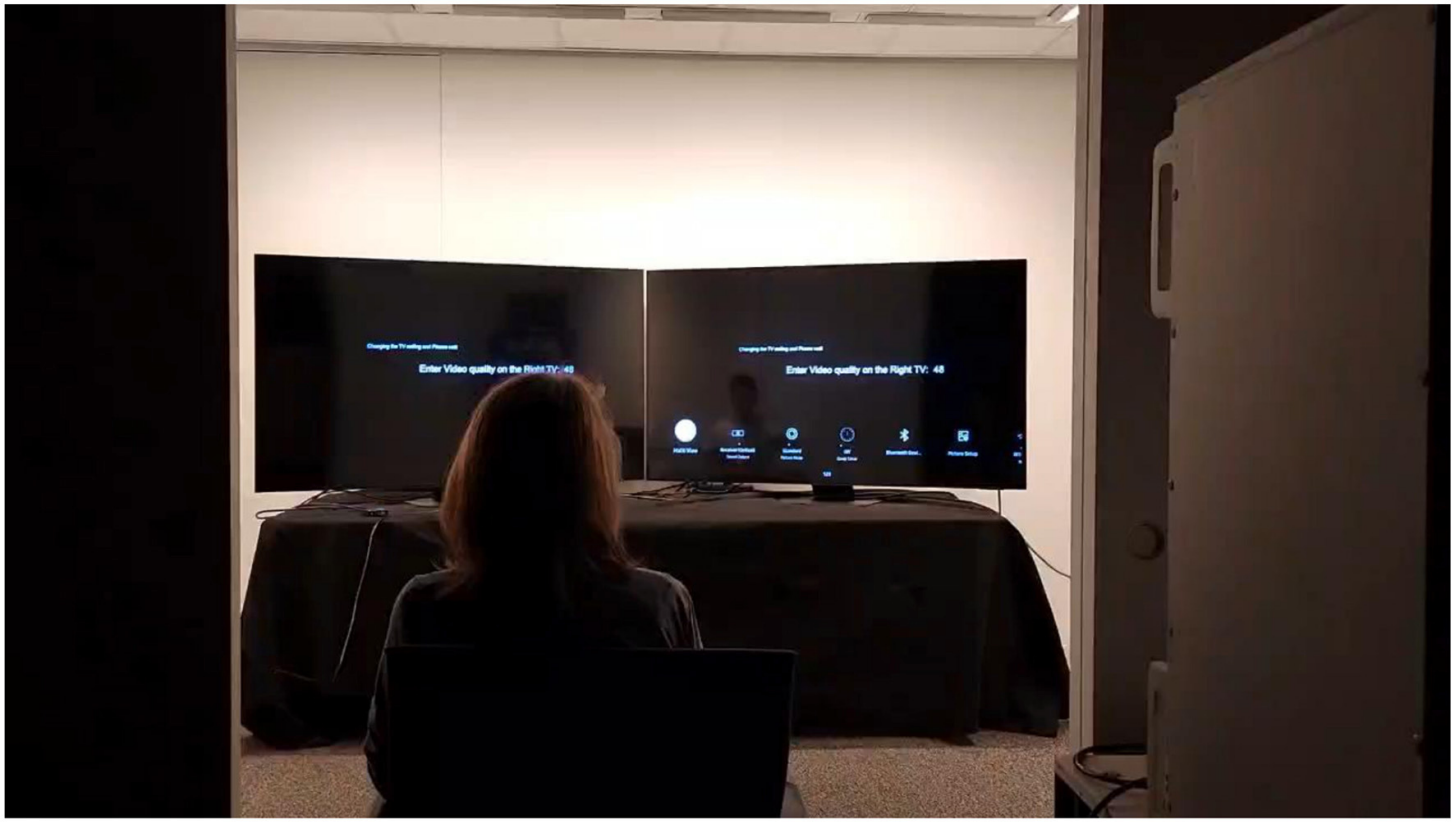
Experimental setup.

A 15-minute warm-up period was allowed for the TVs and the LED ceiling lights. During the experiment, observers sat nine feet from the center of two TV displays, as shown in Figure 3. The viewing angles for both TVs were identical. A one-minute adaptation period between the blocks was required for the observers, allowing them to adapt to the ambient light changes adequately. The video stimuli were set as full-screen. Observers responded to the same content twice within each block.

Considering four ambient lighting conditions, two Picture Mode categories, five visual stimuli, and two repetitions, each observer performed 80 video quality assessments. This required approximately 25 minutes of experimental time per observer.

### 2.4 Experiment II: procedure

The second experiment largely mirrored Experiment I, except that the picture settings were on the colorfulness scale and an additional stimulus was included (a bright movie scene; see Figure 2F). Both TVs were configured with identical picture settings but differed in their colorfulness level. The reference TV employed a baseline (default) colorfulness, while the comparison TV was set to (1) low, (2) medium, and (3) high levels of colorfulness for separate trials.

This experiment consisted of four blocks of different lighting conditions, three distinct colorfulness levels on TV displays, and six stimuli with two repetitions. This section was divided into 12 segments, encompassing 144 trials. The duration of this experiment was approximately 40 minutes per observer. The MATLAB program controlled the entire experimental procedure, similar to the first experiment.

After completing both experiments, we performed a survey to collect observer characteristics (e.g., age, gender) and study participants' qualitative feedback about the experiments.

## 3 Results and discussion

The conducted experiments generated two datasets: 2,960 observations for Experiment I and 5,328 observations for Experiment II.

### 3.1 Analysis of factors

#### 3.1.1 Experiment I

An ANOVA test was conducted on the data using JMP software to analyze the impact of various factors under each experimental condition. These conditions were grouped based on demographic factors, with observer variations considered random. Drawing conclusions from the *P*_*value*_, the significant factors included TV's picture setting, luminous intensity, video content, age, country, and habit. The interactions between picture setting & intensity and picture setting & video content were significant in Experiment I. (The statistical results of Experiment I are in Appendix).

The observer was asked to compare the default picture settings to newly identified, user-preferred ones under two presets (i.e., Standard and Movie Picture Mode). Here, our aim is to understand how people perceive user-preferred Picture Mode compared to the default. To determine the overall preferences of observers toward the given Picture Mode, Experiment I data was segmented by Picture Mode: Standard and Movie. A response exceeding 50 signifies a preference for the user-preferred Picture Mode (e.g., Bright Movie Mode). Other indicates a preference for the reference (default) Picture Mode. A response equal to 50 indicates no preference between the two Picture Modes. Then, two one-sample t-tests were separately applied to the Standard and Movie Mode data. The null hypothesis was that the user-preferred Picture Mode is equivalent to or worse than the default. The t-test results indicated we failed to reject the null hypothesis at the significance level of 0.05 for Standard Mode. However, for Movie Mode, the test suggested to reject the null hypothesis. Therefore, the conclusion is that observers preferred the default picture settings under Standard Mode but preferred the user-preferred settings (increased brightness) for Movie Mode.

**Figure 4:**
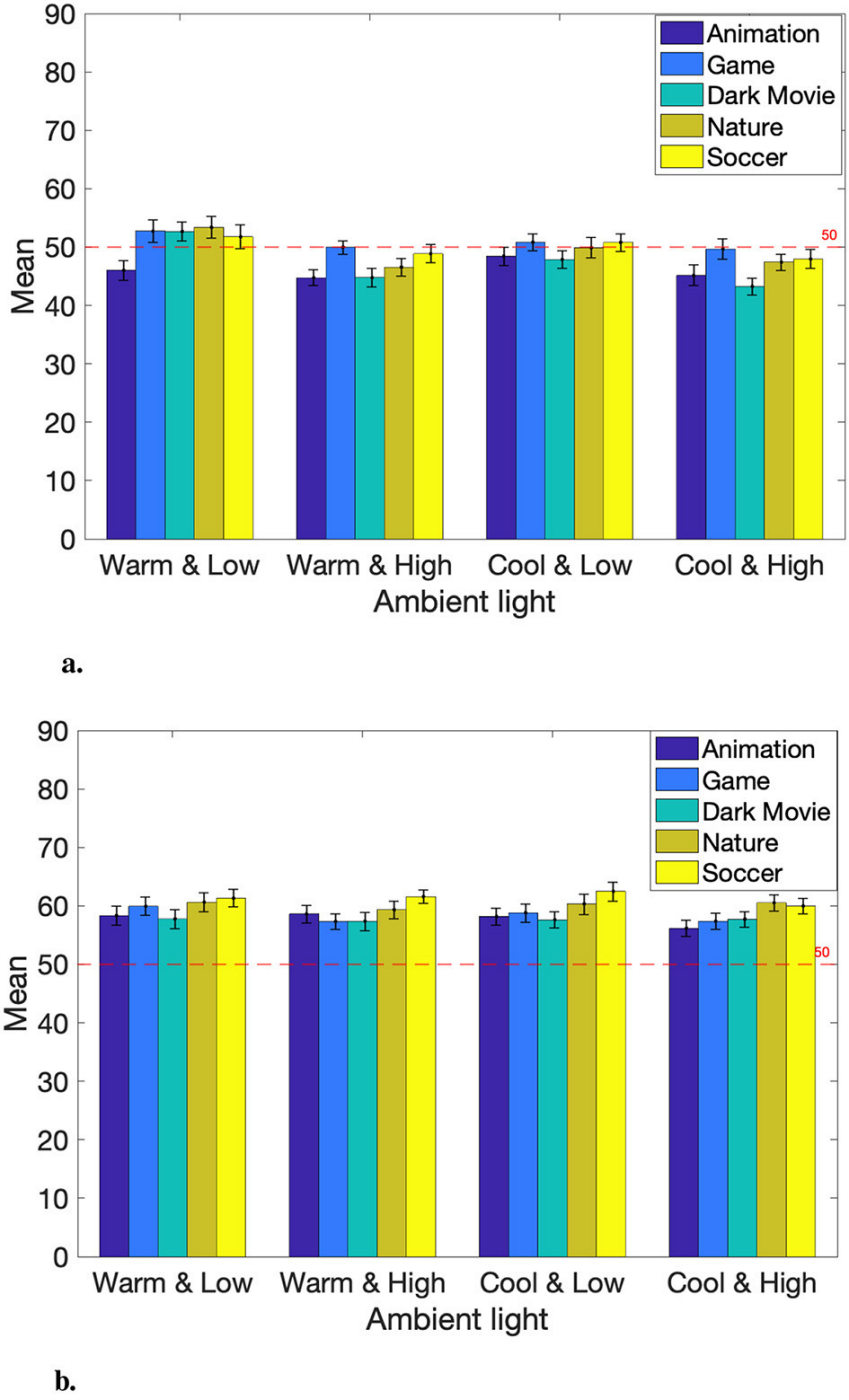
Overall preferences of TV's picture settings. **(A)** Mean responses for user-preferred Dark Standard Mode. **(B)** Mean responses for user-preferred Bright Movie Mode.

The Experiment I data was further divided by ambient lighting conditions and content. There were four lighting conditions: (1) Dark Warm, (2) Bright Warm, (3) Dark Cool, and (4) Bright Cool, and five different visual stimuli: (1) Animation, (2) Game, (3) Movie (Dark), (4) Nature, and (5) Sports. The observers' responses for each video content under specific lighting conditions are presented in Figure 4. Regarding Movie Picture Mode, mean responses consistently exceed 50 regardless of ambient lighting and content (see Figure 4B). This suggests that observers generally preferred the user-preferred (bright) Movie Mode. In contrast, most of the means fall below 50 for the Standard Mode, indicating a preference for the default setting. However, in the case of warm and low-intensity lighting conditions (Dark Warm), observers tended to favor the user-preferred setting (darker than the default Standard) except for Animation content.

Multiple comparison *t*-tests were applied to the Experiment I data to better understand the inter-relationships between ambient lighting and content genre. Table 3 demonstrates the results of the multiple comparison t-tests across different lighting conditions with varying content pairs. Since each lighting condition consists of ten comparison content groups, Bonferroni correction were conducted with $\alpha_{corrected}=\frac{0.05}{10}=0.005$.

**Table 3:** Multiple *t*-tests on content/lighting (Experiment I).

**Content\lighting**	**Dark warm**	**Bright warm**	**Dark cool**	**Bright cool**
Animation vs. game	0.0097	**0.0039**	0.2665	0.0721
Animation vs. movie	0.0057	0.9898	0.7966	0.4061
Animation vs. nature	**0.0045**	0.3747	0.5387	0.3190
Animation vs. sports	0.0336	0.0518	0.2821	0.2499
Game vs. movie	0.9784	0.0089	0.1558	**0.0045**
Game vs. nature	0.8116	0.0668	0.6810	0.3127
Game vs. sports	0.7313	0.5710	0.9949	0.4755
Movie vs. nature	0.7771	0.4175	0.3811	0.0364
Movie vs. sports	0.7336	0.0716	0.1698	0.0306
Nature vs. sports	0.5665	0.2852	0.6924	0.8021

We used dark scenes under the movie genre for Experiment I. Significant *p*-values instances are emphasized (bold-faced).

As can be seen, several circumstances indicate users prefer customized picture settings other than the default. Specifically, our study subjects were more likely to prefer the dark version of Standard Mode when watching Nature content compared to Animation content under the dark and warm room lighting condition (see Animation vs. Nature and Dark Warm in Table 3; *p* < 0.005). The Nature content was a sunset scene with warm colors, so this might be related to the perceived darkness and the observer favoring a darker image tone. The opposite goes for the Movie Mode. The observers preferred the bright Movie Mode when watching cinematic content over game content under the bright and cool room lighting condition (see Game vs. Movie and Bright Cool in Table 3; *p* < 0.005). This finding aligns with some literature: people prefer brighter images on a display device (e.g., TV) in a bright environment and *vice versa* (Lee and Park, 2021; Lee, 2023).

The same analysis was applied between four different lighting conditions in order to investigate the illumination level and CCT factors. The results are listed in Table 4. There are statistically significant differences between Dark Warm and Bright Warm, Dark Warm, and Bright Cool, and Bright Warm and Dark Cool (*p* < 0.0083, Bonferroni-corrected for six comparisons). The results indicate that the room illuminance level has a more substantial effect than the CCT.

**Table 4:** Multiple t-tests on lighting conditions (Experiment I).

**Lighting condition pairs**	***p*_*value*_**
Dark warm vs. bright warm	**0.00**
Dark warm vs. dark cool	0.3577
Dark warm vs. bright cool	**0.0058**
Bright warm vs. dark cool	**0.0011**
Bright warm vs. bright cool	0.1923
Dark cool vs. bright cool	0.0582

Significant *p*-values instances are emphasized (bold-faced).

The significant demographic factors were age, country, and habit. For the age factor, the older group gave significantly higher scores than the younger group toward the newly discovered Picture Mode. The analysis of the interaction effect of Picture Mode and age suggested that the older group preferred the Dark Standard Mode, while the younger group did not like the Dark Standard Mode. For the Movie Mode, both groups preferred the Bright Movie Mode. Participants with North American/European backgrounds had a lower preference score for new Picture Modes than participants from other regions. The night-time watchers also gave lower scores for non-default Picture Modes than other time watchers.

#### 3.1.2 Experiment II

An ANOVA test was applied to the data collected for Experiment II. The test results indicate the video content, age, country, habit, and gender were the significant factors. The interaction of picture setting & CCT was significant, too. We implemented and installed specialized software on the control TV to show distinct levels of colorfulness (low, medium, high) while controlling other image quality attributes (e.g., brightness). However, while preparing Experiment II, we realized that the perceptual difference between the reference and control TVs was relatively less noticeable compared to Experiment I. This might yield slightly different results about impacting factors. (The statistical results of Experiment II are in Appendix).

**Figure 5:**
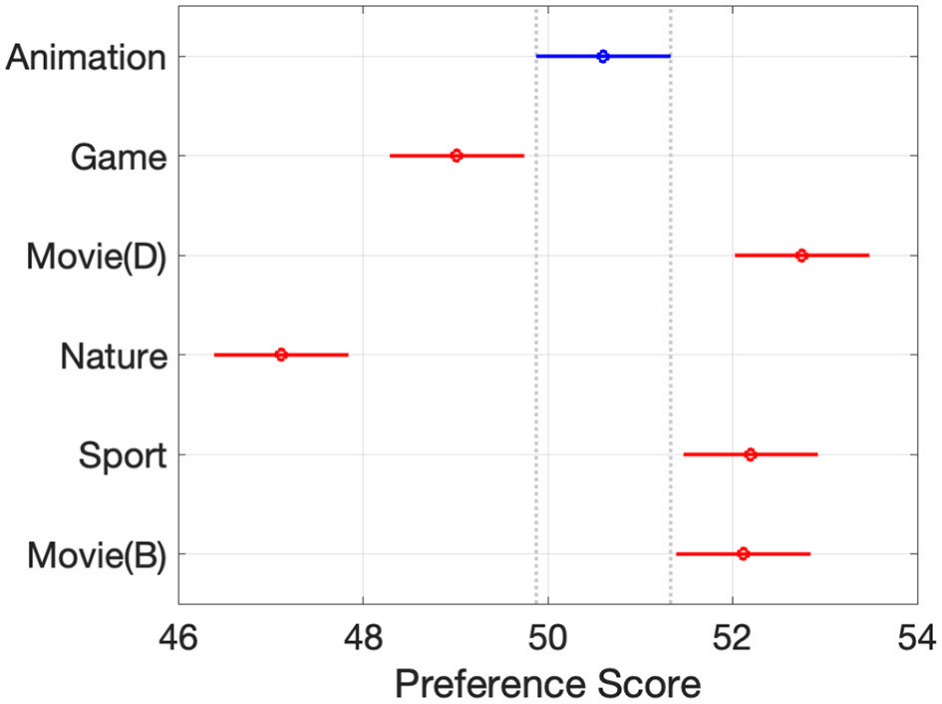
Responses per content.

The observers' responses grouped by the video content factor are shown in Figure 5. Animation and Game content were significantly different from other content; they were both animated scenes, which might make them stand out. It also indicates that Movie (Dark), Sports, and Movie (Bright) content were similar to each other and distinct from others. The commonality between these videos was that they all included skin tones.

To probe whether the genre itself or the specific video content was the driving factor, the Bright Movie clip was included in Experiment II. A two-sample t-test was used to investigate differences in viewer preferences toward Movie (Dark) and Movie (Bright) content under a single, bright TV viewing environment. The null hypothesis was that there exist no differences in user preferences toward dark and bright scenes from the same movie content (see Figures 2C, F). The test rejected the null hypothesis at the 0.05 significance level. Therefore, people perceive picture quality differently, even in the same video, so the current genre categorization may not effectively capture people's actual perceptions and preferences.

Similar to Experiment I, the demographic factors of age, country, and habit were again found to be significant. Moreover, adding colorfulness manipulation revealed the significance of the demographic factor of gender. The interaction between colorfulness and gender, along with the gender factor itself, was examined. Recall that the test TV was equipped with a color enhancement algorithm. The lowest colorfulness level for this TV was reasonably equivalent, although not a match, to the colorfulness of the reference TV. For the medium level of the colorfulness setting, both genders gave scores above 50 indicating that the participants generally prefer more colorful rendering. Overall, women gave lower scores than men observers, who gave scores higher than 50, regardless of the colorfulness level. The younger group gave a higher score than the older group showing a consistent preference for the enhanced colorfulness, while the older group showed a preference for the default. The North America/Europe group also showed a preference for the reference TV, but the other group preferred higher colorfulness levels. Night-time watchers gave lower scores toward the enhanced colorfulness than others.

## 4 Appearance based analysis

The observers' feedback was collected to review how they evaluated the videos. According to the feedback, the descriptors commonly used were brightness, chroma, naturalness, skin tone, and grass color. These words suggested that some observers judged by overall scene color attributes, and others were more focused on object colors, such as skin tone and grass colors. Two color appearance-based analyses were conducted to get insight into the correlation between preferences and these factors. The color attributes and the representative colors of the objects in the videos were analyzed.

### 4.1 Color attributes analysis

The keyframes of the videos were selected and saved in RGB format. Then, a display characterization model was used to convert the display value (RGB) to the tristimulus values (XYZ). With the aid of the image appearance model, the tristimulus values were converted to color attributes, which were used for further analysis.

#### 4.1.1 Display characterization models

This project examined TV displays with eight distinct settings along an ambient environment variation containing four unique lighting conditions. This resulted in a total of 32 combinations for the analysis of TV settings and ambient conditions. For the display characterization, the PR655 spectroradiometer was used to measure the radiance of different color ramps, and afterward, these measurements were converted into absolute XYZ values. Consequently, 32 distinct display characterization models were created for different conditions. The detailed transformation sequence can be found in Berns (1996). Each display model corresponds to a specific combination of picture settings and ambient light, ensuring the capture of the display's color rendering capabilities under varied environmental lighting. For display characterization, the display model was applied, which is based on the principles of additivity and scalability. For each display model, the test results showed that the model could predict color with the mean error in the range of 2 to 5 units of Δ*E*2000. Additionally, the display uniformity was assessed by measuring a set of colors, specifically red, green, blue, and a randomly chosen color, lime. There were five measurements for each color: one at the center and four at the corners of the display. The calculated DE00 values for these measurements were 1.27, 0.84, 0.56, and 0.89, respectively, with an average of 0.89. The uniformity of the display was within the satisfactory level. The display model conducted around the center area of the display could represent the whole display.

#### 4.1.2 Image appearance attributes

In Experiment I, five videos were used for analysis (see Figure 2). Keyframes from these videos were extracted and saved as RGB PNG files. The RGB values were then converted to *XYZ*_*im*_ values using the display models. The *XYZ*_*im*_ values obtained were the absolute values and were used as the basis for further transformations or normalization.

To standardize the data, a chromatic adaptation model was used to convert these *XYZ*_*im*_ values under a *XYZ*_*D*65_ white point. Given that the experimental setup involved two TVs side-by-side with ambient lighting, three distinct white points were present: one for each TV and one for the ambient light. The white point for adaptation was chosen based on the brightest one for each combination. The chromatic adaptation is as follows:


(1) $$\begin{array}{l} XYZ_{D65}=M_{16}^{-1}\times M_{adp}\times M_{16}\times XYZ_{im} \end{array}$$

where *M*_16_ is from CIECAM16 (Li et al., 2017), *M*_*adp*_ = diag(*D*65./*White*_*scene*_), represents the adaptation matrix to convert scene white point(*White*_*scene*_) to D65. Full adaptation is assumed.

#### 4.1.3 Image appearance analysis

Traditional image-based analysis considers the pixel-wised values, and frequently, it does not directly incorporate any of the spatial or temporal properties of human vision and the perception of the image. This might miss some important concepts in image/video quality perception. Winkler (1999) discussed the importance of the video quality related to vision. Soundararajan and Bovik (2012) considered the spatial factors to qualify the video qualities, which shows promising results. The image appearance model (iCAM; Fairchild, 2013) was used to aid the analysis. The image appearance parameters were assessed by using an iCAM in IPT space, which included lightness (I), chroma (C), and hue (h). The contrast sensitivity function (CSF) was utilized to filter the images. Three filters were applied to the luminance and two chromatic channels. The filters are calculated as follows:


(2) $$\begin{array}{l} csf_{lum}=a\times f^{c}\times e^{-b*f} \end{array}$$

(3) $$\begin{array}{l} csf_{chroma}=a_{1}\times e^{-b_{1}*f^{c_{1}}}+a_{2}\times e^{-b_{2}*f^{c_{2}}} \end{array}$$

The filters are operated in IPT color space. Fourier transformations are used. *csf*_*lum*_ is applied to I channel, and two *csf*_*chroma*_ are applied to P and T channels. The parameters' value can be found in Fairchild (2013). And *f* is defined in terms of cycles per degree of visual angle (cpd), which is


(4) $$\begin{array}{l} f=\frac{ppi}{\frac{190}{\pi }\times tan^{-1}\left(\frac{1}{dis}\right)} \end{array}$$

where *ppi* is pixels per inch of the display, and *dis* is the distance between the observer and the display.

In the keyframe analysis, two TVs were assessed: one as a reference and the other as the test display. Differences in appearance attributes were quantified, including lightness difference (Δ*I*) and chroma difference (Δ*C*).

### 4.2 Color attributes in CIELAB

A comparison was conducted between the IPT-based image appearance model and CIELAB space. In CIELAB space, the L^*^a^*^b^*^ values were calculated after chromatic adaptation, with the same *XYZ*_*D*65_ as were used for the IPT calculations with D65 as the white point. Both the lightness (L) and chroma (C) values were derived from these coordinates. Similar to the approach with IPT, differences in lightness (Δ*L*) and chroma (Δ*C*) were calculated and then compared to the mean preference.

#### 4.2.1 Results

**Figure 6:**
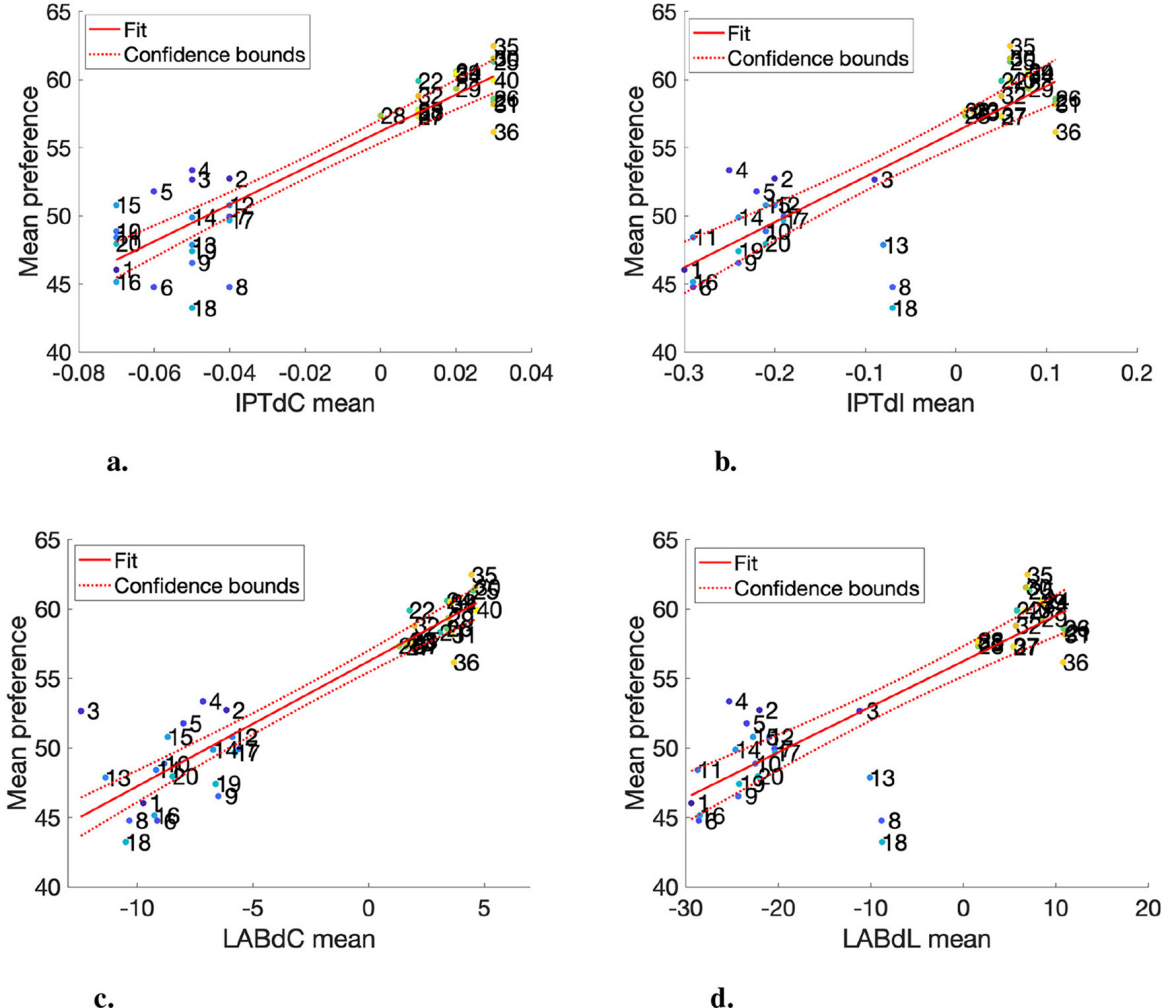
Mean chroma and lightness with the mean preference response in IPT color space and CIELAB. **(A)** Mean IPTdC and mean response (*R*^2^ = 0.86). **(B)** Mean IPTdI and mean response (*R*^2^ = 0.71). **(C)** Mean LABdC and mean response (*R*^2^ = 0.85). **(D)** Mean LABDL and mean response (*R*^2^ = 0.73).

Figures 6A, B present the results obtained from Experiment I. Each data point represents the mean preference response of a specific video content assessed under a particular lighting condition (see Table 5). In these plots, the y-axis represents the mean preference response of the observer, while the x-axis indicates the Δ values for various appearance attributes. The data points in these plots tend to be clustered into two distinct groups: Standard and Movie Picture Mode. Notably, in the Movie Mode, observers generally show a preference for the test configuration (i.e., Movie Mode with increased brightness), whereas in the Standard Mode, the default one tends to be favored.

**Table 5:** Video index table (Experiment I).

**Content\lighting**	**Dark warm**	**Bright warm**	**Dark cool**	**Bright cool**
Animation	1, 21	6, 26	11, 31	16, 36
Game	2, 22	7, 27	12, 32	17, 37
Movie (dark)	3, 23	8, 28	13, 33	18, 38
Nature	4, 24	9, 29	14, 34	19, 39
Sports	5, 25	10, 30	15, 35	20, 40

1–20 were shown under standard picture mode. 21–40 were shown under movie picture mode.

The regression analysis is applied to determine how lightness and chroma influence preference. The regression models and their confidence bounds are plotted in Figures 6A, B. The models have a reasonable performance with *R*^2^ values of 0.86 and 0.71, respectively. Importantly, the coefficients for both models are positive, implying a linear relationship between lightness and chroma and preference so that as the lightness and chroma within the range of our data increase, preference appears to increase correspondingly.

The analysis based on the image appearance model shows a clear trend. The conclusion drawn from this analysis is that observers tend to prefer images that are brighter and more chromatic. This preference pattern is consistent across the different picture settings in Experiment 1. Resulting in given videos under four unique lighting conditions following a similar preference order: Dark Warm, Dark Cool, Bright Warm, and Bright Cool conditions. As an example, data points 3, 13, 8, and 18, which are the Movie (Dark) content under the four lighting conditions. The results are aligned with the Bonferroni test in terms of lighting conditions, in which observers prefer the dimmer ambient lights.

As shown in Figure 6, the comparison of color spaces reveals that the lightness difference and preference plots between the two spaces are almost identical. However, the chroma plots, while overall trending similarly, differ significantly in the order of chroma differences (IPTdC and LABdC). As an example, the third image under warm and dim lighting (Index: 3) has the lowest chroma difference in CIELAB, but not in IPT. In comparing IPT and CIELAB, it is evident that the IPT image appearance model accounts for the contrast sensitivity of human vision, whereas CIELAB evaluates color on a pixel-by-pixel basis, which is closer to traditional image difference calculations. The chroma difference and preference plots indicate that as chroma difference increases, so does preference. The IPT model more effectively captures this trend; data point 3 specifically shows a relatively higher preference and aligns more closely with the regression line in IPT than in CIELAB. Thus, while the preference trend relative to lightness changes is similar between the two spaces, IPT better captures chroma variations.

### 4.3 Representative color analysis

**Figure 7:**
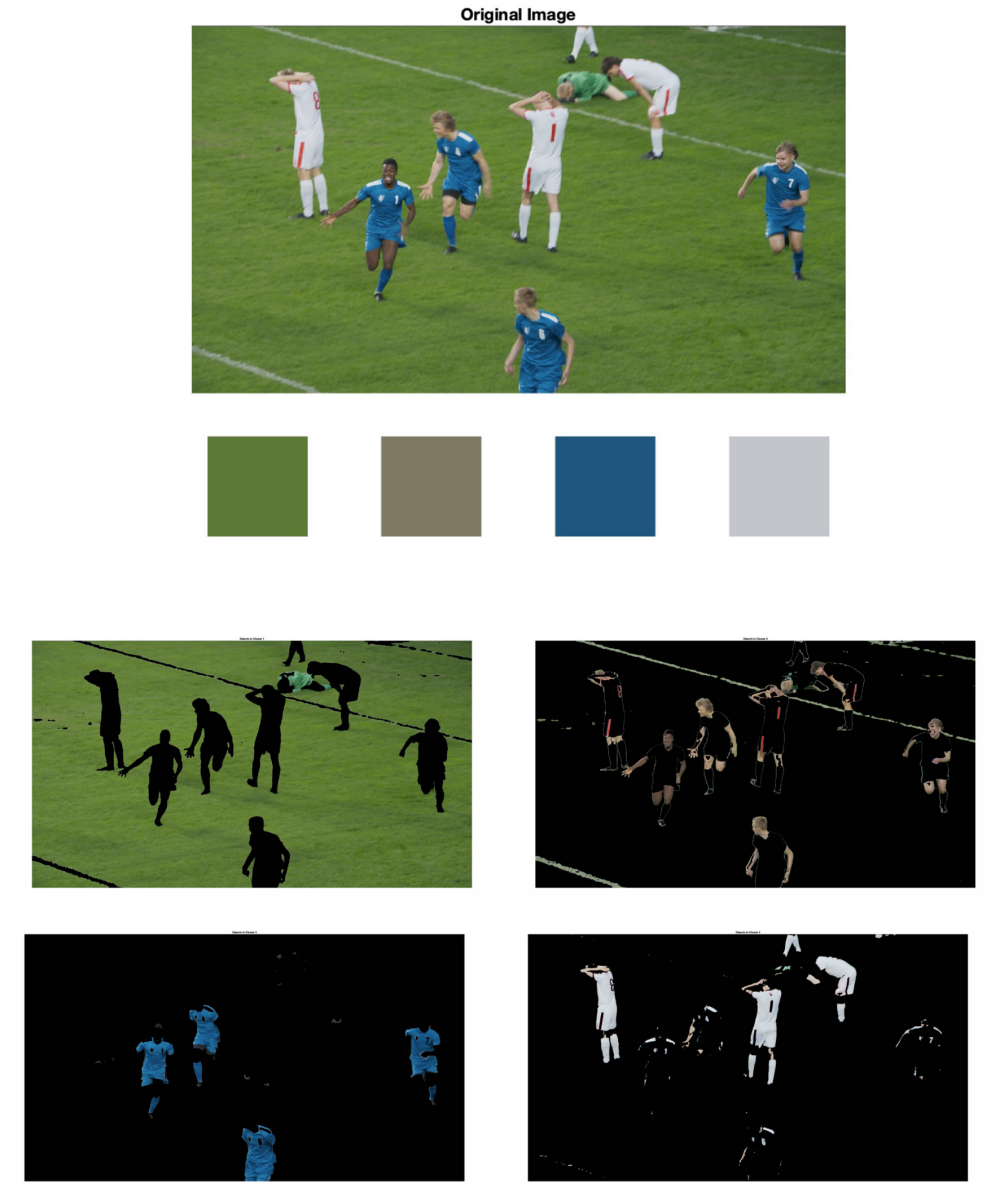
Color segmentation results (sports content).

Image segmentation has been used for the analysis of the image's features, including classification or color enhancement (Comaniciu and Meer, 1997; Naccari et al., 2005). In this section, image segmentation was used to analyze the visual appearances of the test content. Initially, each image was transformed into the CIELAB color space. Following this, a *K*-means clustering algorithm was applied to segment the images in CIELAB space, setting *K* to four. This segmentation aims to isolate four principal colors in each image. They are aligned with the objects' colors that the observers used for evaluating their preferences. The presentation sequence started with the original image, followed by the four representative colors, which were computed by averaging the colors within their respective areas. The subsequent four images display the individual areas associated with each of these representative colors. As an example, Figure 7 shows four segmented areas representing the grass, skin tones, and two uniforms. The other images follow the same fashion.

For this study, a total of 32 display models were generated, each corresponding to a unique combination of the TV's picture setting and ambient lighting conditions. These display models converted the representative colors from RGB to XYZ. Subsequently, CIECAM16 was applied to convert these XYZ values to the D65 white point. The white luminance was the absolute white in *cd*/*m*^2^, and adapting luminance was set to 20% white luminance and the average condition in CIECAM16 with full adaptation. The average condition was used because the luminance of the white in the scene was always above 200 *cd*/*m*^2^. The color appearance attributes were then calculated, and the differences in the attributes were determined. The CIELAB color space was used to analyze color appearance attributes, with calculations based on the *XYZ*_*D*65_ tristimulus values, which used chromatic adaptation to the D65 white point. Following this, differences in color attributes were determined. The results from CIELAB were then compared with those from CIECAM16. The analysis then focused on correlating these appearance attributes with the viewers' preference responses for each unique experimental condition.

**Figure 8:**
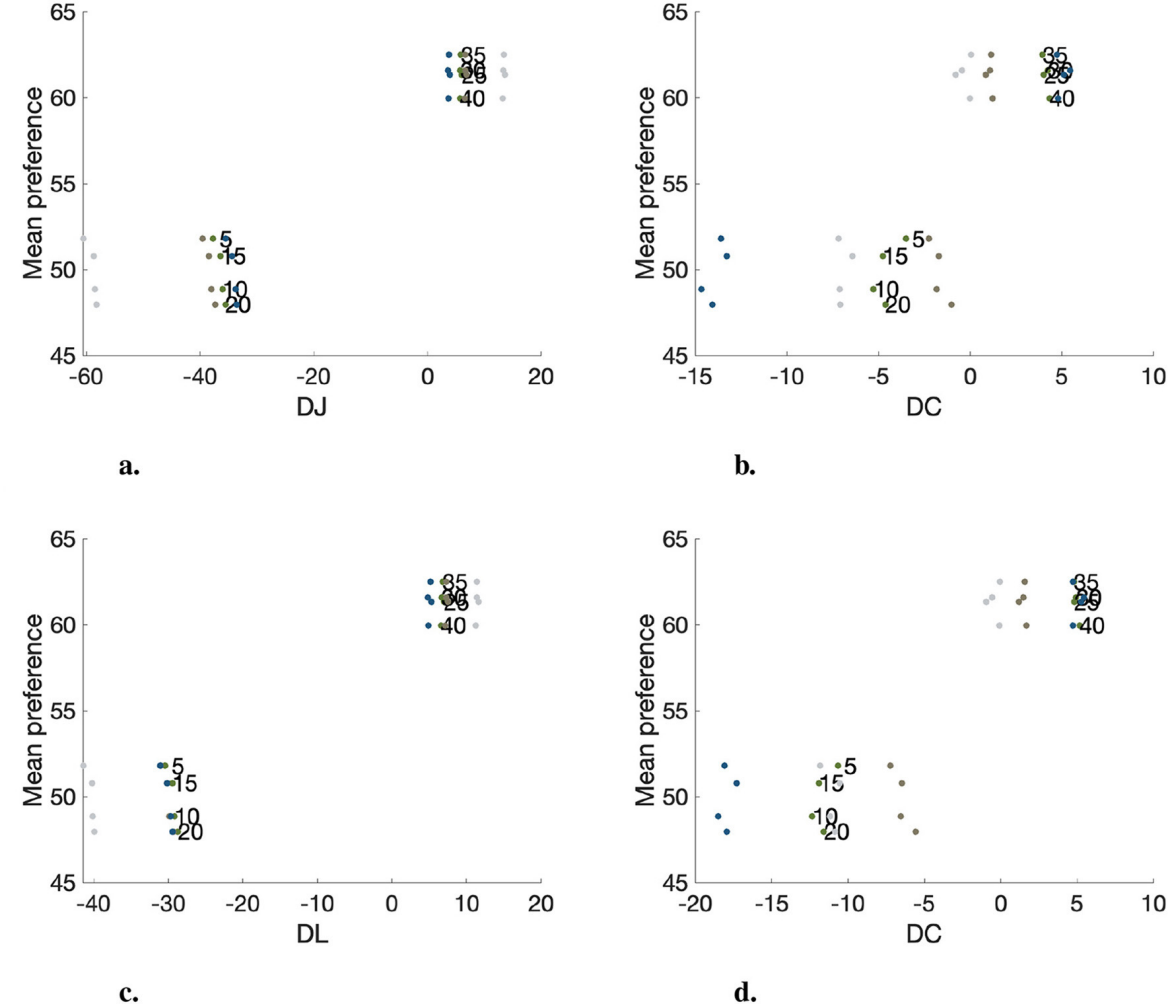
Mean preference per lightness and chroma of representative colors in CIECAM16 and CIELAB. **(A)** Lightness plot of sports content in CIECAM16. **(B)** Chroma plot of sports content in CIECAM16. **(C)** Lightness plot of sports content in CIELAB. **(D)** Chroma plot of sports content in CIELAB.

The attributes of the represented colors were calculated using CIECAM16 and CIELAB and used for analysis. Since each video features distinct representative colors, the data were categorized and analyzed according to the individual videos. Figure 8 depicts how the preference rating changes according to the lightness and chroma of four representative colors in Sports content (soccer scene). The index numbers remain consistent with those used in the image appearance attribute analysis (see Table 5). To enhance clarity and facilitate interpretation, the color of each dot matches the actual color of the represented area within the video. As confirmed in the appearance-based analysis, for a given representative color, the brighter and more chromatic the color, the more observers preferred the video. Overall, both color spaces provide similar insights when comparing differences in color attributes and preferences. However, the magnitudes of four representative colors under the same lighting condition show greater variance with CIECAM16 compared to CIELAB. For example, for Index 5, which represents the image 5 under warm and dark conditions, Figure 8C reveals that only one representative color, the lightest color shown in Figure 7, displays a larger magnitude of color differentiation in CIELAB, with the rest grouped closely together. In contrast, CIECAM16 exhibits better separation for all representative colors, likely due to its more thorough consideration of environmental factors including illumination level. Further investigation is needed to compare the two spaces more extensively.

### 4.4 Common representative colors

**Figure 9:**
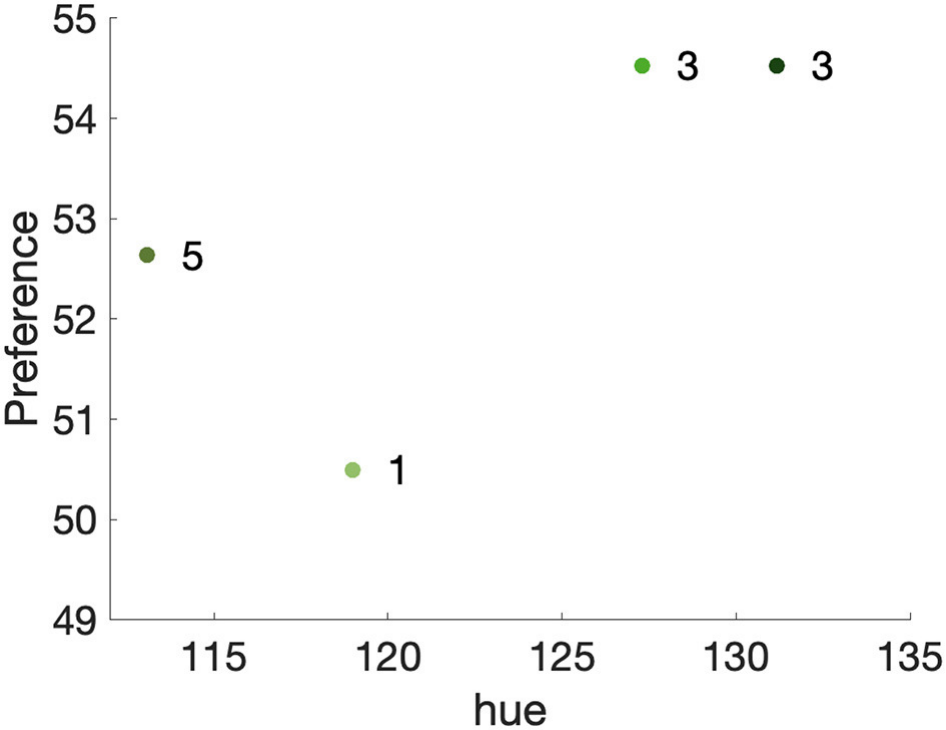
Preference per hue. Green represents the color of animation, movie (dark), and soccer content under the same picture setting and lighting condition. The x-axis is the hue of different shades of green on the test display.

There are several similar representative colors among the content used in our experiments. Specifically, Animation, Movie (Dark), and Sports content all has green as one of the representative colors (see Figures 2A, C, E, respectively). The hue angles for these greens were calculated in the CIECAM16. The hue angle ranged between 113° and 131°. The hue differences are within 20°, as shown in Figure 9. The green hues in different items, an animated green tree, grass in the soccer field, and a green block, have similar color attributes (see Table 6), but the preferences of these videos were very different. An interesting aspect to consider is the context in which the green color appears. In movie (Dark) and sports content, green is a part of real-life objects, while in animation content, it is part of animated scenes. Even though similar shades of green were presented to the observers, their preferences significantly varied between real-life and animated footage. Similarly, Animation, Game, and Nature content all contain blue sky (see Figures 2A, B, D), but Animation and Game content are both animated, and Nature content is a real scene. As a result, the preference for Nature content was significantly different from that for Animation and Game content. Even when blue was ranked as the second prominent representative color for Game and Nature content, their preferences are significantly different from each other.

**Table 6:** Differences in attributes of green representative color.

**Video**	***ΔLightness***	***ΔChroma***	***ΔSaturation***	***Δhue***
Animation	5.20	1.94	0.43	-2.82
Movie (dark) 1	5.55	3.92	0.37	-0.38
Movie (dark) 2	2.15	2.87	0.64	-0.24
Sports	3.02	0.08	-0.73	-2.42

The calculation is based on the CIECAM16.

All the comparisons above indicate that the substantial differences in content lie in whether the scene was animated or captured in real life. Therefore, the naturalness of the videos and observers' memory color of specific objects (e.g., grass) affect people's video quality preferences.

## 5 General discussion

This project was designed to evaluate the influence of various factors on people's video quality preferences. Our findings suggest that TV picture settings, the intensity of ambient light, the video content, age, country, habit, and gender have significant effects on TV viewers' preferences.

In our experimental design, the illumination level of ambient light compared to that of the TV was generally lower (15–350 lux). This could potentially diminish the influence of correlated color temperature (CCT) on the study subjects' responses.

The appearance- and color-based image analyses per each experimental condition were conducted to get a deeper insight into video quality preference determinants. The results of the analysis indicate that chroma, lightness, and memory colors are essential in understanding people's preferences. In general, people prefer brighter and more chromatic images. Moreover, memory colors, such as grass and skin tones, significantly influence their perception and preferences of the visual quality of displayed content.

For display characterization, a display model based on the principles of additivity and scalability was applied. Under the same picture setting, the assumption of additivity and scalability was not strictly valid, but acceptable. Additionally, the display uniformity was assessed and found to be within a satisfactory level. Overall, the variations observed were unlikely to influence our results significantly.

**Figure 10:**
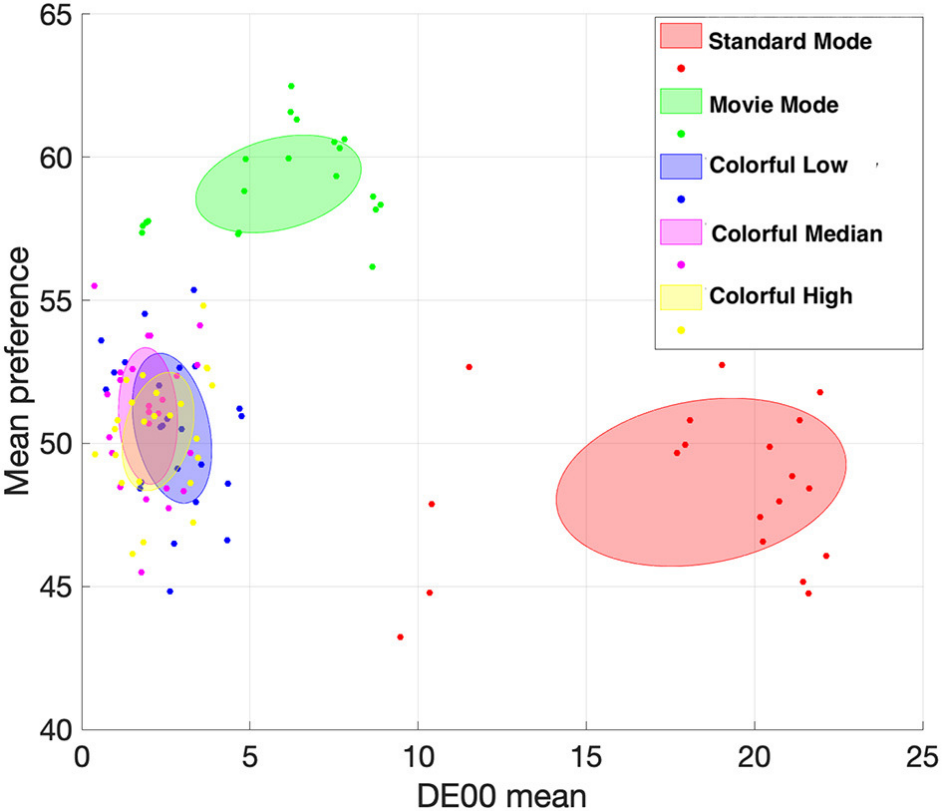
Mean preference and DE00 variation. This figure illustrates the preference differences among various TV modes: Standard, Movie, and Colorful. The ellipses represent the variance-covariance of preferences. While clear differences are observed between the Standard, Movie, and Colorful modes, the preferences within the various levels of colorful mode show significant overlap.

The gender factor was significant in Experiment II, but not in I. The difference between the two experiments was the magnitude of changes in color attributes (i.e., colorfulness level). The two experiments' color differences (DE00) and mean preference results are shown in Figure 10. By analyzing the content used in Experiment I, regarding the differences in lightness and chroma, we state that people prefer brighter and more chromatic scenes, in general. We focused on colorfulness in Experiment II. Even if we experimented with three different colorfulness levels, the perceptual differences between the reference and control TV (enhanced colorfulness) were smaller than that of Experiment I. Still, some observers reported that they perceived noticeable changes in the naturalness of skin tones for content involving human faces. These analyses suggest that there exist no meaningful gender differences in perceptual visual quality in terms of substantial lightness and chroma changes that were employed in Experiment I. In contrast, viewers' gender could impact their perception of video content formatted with smaller changes in the colorfulness levels (Experiment II). Moreover, our data shows that the preference for larger differences is similar for all genders, but varies for small color differences, potentially indicating a gender-based difference in preference sensitivity to small changes in color rendering.

The image-based analysis provided a comparison of color spaces, specifically between the IPT image appearance model and CIELAB. The IPT model accounts for contrast sensitivity of human vision, while CIELAB calculates differences on a pixel-by-pixel basis. While the lightness difference and preference scores are similar in both color spaces, IPT is promising in representing chroma and other color attributes, capturing the trends more effectively in displayed images. Conversely, CIELAB, though more straightforward to implement and performing well with chromatic adaptation, does not represent chroma as effectively as IPT. Therefore, the IPT image appearance model offers better representation of certain color attributes, such as chroma. Further research is needed to explore the differences in color attributes and preference scores more thoroughly. Moreover, a color space comparison between CIECAM16 with CIELAB was conducted as part of the representative color analysis. The results indicate that while both spaces showed very similar results for the preference data, CIECAM16 demonstrated better differentiation. This might be due to CIECAM16's consideration of environmental factors. This aspect requires further exploration to understand the implications more thoroughly.

The effectiveness of using genre to categorize video types needs to be examined. For instance, our results indicate a discernible difference in viewer preferences between animated and real-life scenes. This suggests that the content within these scenes is significantly independent from the genre itself. Another example is the presence of skin tones (Anku and Farnand, 2020; Farnand and Fairchild, 2014), which emerged as a critical feature in video content regardless of the genre. Observers' memory of the grass and the sunset, which in this case are from different genres, is the key to the preference—how the naturalness of these colors aligns with the viewer's memory.

## 6 Conclusion

In this article, we conducted a comprehensive analysis of how individuals perceive visual quality on television (TV) displays. Our study took into account various factors such as ambient lighting, display settings, viewing content, and personal characteristics. Our findings indicate that the intensity of room illumination, type of video content, and picture settings significantly affect TV viewers' picture quality perceptions and preferences. User-specific factors like age, country of origin, TV viewing habits, and gender also play a role in determining video quality perception, highlighting the need for personalized image/video rendering. Our research also suggests that the conventional categorization of content genres may not be a reliable basis for content-based video enhancement. We found that people perceive video quality differently based on the characteristics of each scene, even within the same content. Furthermore, our results indicate that people generally prefer brighter and more chromatic videos, with natural object colors and memory colors (such as skin tones and grass) having a significant impact on their preferences.

## Data Availability

The raw data supporting the conclusions of this article will be made available by the authors, without undue reservation.
