# Supplementary material for: Factors impacting human-perceived visual quality on television displays
Source: Front Neurosci. 2024 Dec 19;18:1426195. doi: 10.3389/fnins.2024.1426195 (PMC11694261; doi:10.3389/fnins.2024.1426195)
Supplement: Supplementary file 1 [file Table_1.docx]

**Experiment I : Fixed Effects Tests**

| **Source** | **Nparm** | **DFNum** | **DFDen** | **F Ratio** | **Prob > F** |
| --- | --- | --- | --- | --- | --- |
| Video Content | 4 | 4 | 1451.0 | 5.57 | 0.0002* |
| Picture setting | 1 | 1 | 1451.0 | 298.81 | <.0001* |
| CCT | 1 | 1 | 1451.0 | 1.22 | 0.2691 |
| Luminance Intensity | 1 | 1 | 1451.0 | 14.23 | 0.0002* |
| Video Content*Mode | 4 | 4 | 1451.0 | 1.54 | 0.1869 |
| Video Content*CCT | 4 | 4 | 1451.0 | 0.18 | 0.9469 |
| Video_ID*Intensity | 4 | 4 | 1451.0 | 0.22 | 0.9274 |
| Picture setting*CCT | 1 | 1 | 1451.0 | 0.36 | 0.5476 |
| Picture setting*Luminance Intensity | 1 | 1 | 1451.0 | 4.87 | 0.0276* |
| CCT*Luminance Intensity | 1 | 1 | 1451.0 | 0.20 | 0.6517 |
| Gender | 2 | 2 | 1451.0 | 0.44 | 0.6444 |
| Age Category | 1 | 1 | 1451.0 | 4.34 | 0.0375* |
| Country Mod | 1 | 1 | 1451.0 | 12.36 | 0.0005* |
| Habit Mod | 1 | 1 | 1451.0 | 70.06 | <.0001* |
| Expertise | 1 | 1 | 1451.0 | 0.20 | 0.6544 |

*Indicates that the factor is statistically significant.
