# Supplementary material for: Factors impacting human-perceived visual quality on television displays
Source: Front Neurosci. 2024 Dec 19;18:1426195. doi: 10.3389/fnins.2024.1426195 (PMC11694261; doi:10.3389/fnins.2024.1426195)
Supplement: Supplementary file 2 [file Table_2.docx]

**Experiment II: Fixed Effects Tests**

| **Source** | **Nparm** | **DFNum** | **DFDen** | **F Ratio** | **Prob > F** |
| --- | --- | --- | --- | --- | --- |
| Video Content | 5 | 5 | 2628.0 | 24.41 | <.0001* |
| Picture setting (colorfulness) | 2 | 2 | 2628.1 | 1.19 | 0.3029 |
| CCT | 1 | 1 | 2628.0 | 2.12 | 0.1452 |
| Intensity | 1 | 1 | 2628.0 | 1.15 | 0.2831 |
| Video Content*Picture setting | 10 | 10 | 2628.1 | 0.80 | 0.6248 |
| Video Content*CCT | 5 | 5 | 2628.0 | 0.37 | 0.8706 |
| Video Content*Luminance Intensity | 5 | 5 | 2628.0 | 0.80 | 0.5473 |
| Picture setting*CCT | 2 | 2 | 2628.1 | 2.63 | 0.0724 |
| Picture setting*Luminance Intensity | 2 | 2 | 2628.1 | 0.85 | 0.4260 |
| Age Categories | 1 | 1 | 2629.8 | 57.32 | <.0001* |
| Gender | 2 | 2 | 2628.2 | 6.49 | 0.0015* |
| Country Mod | 1 | 1 | 2630.0 | 28.52 | <.0001* |
| Expertise | 1 | 1 | 2629.8 | 0.13 | 0.7210 |
| Habit Mod | 1 | 1 | 2629.6 | 11.40 | 0.0007* |

*Indicates that the factor is statistically significant.
